# Perception of Parents of Thalassemic Child to Thalassemia in Pakistan

**DOI:** 10.7759/cureus.17615

**Published:** 2021-08-31

**Authors:** Noman Saleem, Adeel Anwar, Noor Ul Ain Shahid, Rabia Saleem, Zara Saleem, Hannan Asghar, Musab Zubair, Humna Ellahi, Sheel Iqbal

**Affiliations:** 1 Forensic Medicine, Sahiwal Medical College, Sahiwal, PAK; 2 Emergency Medicine, Memorial Hermann Hospital, Houston, USA; 3 Medicine, Ameer ud Din Medical College, Lahore, PAK; 4 Neurosurgery, Punjab Institute of Neurosciences, Lahore, PAK; 5 Plastic Surgery, Lahore General Hospital, Lahore, PAK; 6 Internal Medicine, Orange Park Medical Center, Orange Park, USA; 7 Medicine, Sahiwal Medical College, Sahiwal, PAK; 8 Psychiatry and Behavioral Sciences, Sahiwal Medical College, Sahiwal, PAK; 9 Research and Academic Society, Sahiwal Medical College, Sahiwal, PAK

**Keywords:** thalassemia major, awareness, prevention, screening, transmission

## Abstract

Background

Thalassemia is an inherited blood disorder characterized by reduced hemoglobin synthesis. Aim of our study is to assess the parental knowledge of thalassemia patients and their awareness regarding treatment and preventive measures against thalassemia.

Methods

It is an observational study done at Ali Zaib Foundation Thalassemia Center in Sahiwal, Pakistan, in May 2019. One hundred parents were enrolled in this study and a subjective questionnaire was used to collect data through direct structured survey method over a period of 30 days.

Results

There were parents of 62 (62%) male patients and 38 (38%) female patients, with a median age of 8.5 ± 6.2 years. Forty-three (43%) parents were illiterate while eight (8%) parents were highly educated. Sixty-six (66%) patients were born to parents with consanguineous marriages. Eighty-two (82%) parents were aware of thalassemia, 72 (72%) were aware of the risk of thalassemia due to cousin marriages, 76 (76%) parents were aware of the importance of prenatal diagnosis (PND), while 88 (88%) believed that a PND was beneficial. Fifty-two (52%) parents knew about thalassemia treatment, 80 (80%) were aware of the importance of blood screening, and 14 (14%) patients were receiving iron chelation therapy. Seventy-eight (78%) parents were aware of thalassemia prevention. All parents believed that the public requires awareness of the importance of premarital screening and PND.

Conclusion

Parental awareness regarding β-thalassemia, its treatment and prevention is fair but far from ideal. Premarital screening, provision of accurate information to the public by professionals, and adequate screening and PND of at-risk families can significantly reduce the rates of thalassemias.

## Introduction

Thalassemias are inherited blood disorders defined by reduced hemoglobin (Hb) production. These disorders are of two main types: alpha (α) thalassemia and beta (β) thalassemia. Thalassemias are prevalent in Italian, Greek, Middle Eastern, South Asian, and African populations [1]. Alpha and beta-thalassemias are often transmitted in an autosomal recessive pattern. In order for a child to have thalassemia, both parents must be carriers. There is a 25% risk for each pregnancy resulting in a child with hemoglobinopathy if both parents are carriers of the thalassemia trait. In 2015, thalassemia was reported in 280 million people worldwide and resulted in 16,800 deaths [2,3].

Important strategies to prevent thalassemias include the provision of up-to-date and accurate information to healthcare professionals and the public, premarital public screening, and appropriate counselling and correct detection of families. These have been effective in thalassemia-prevalent countries, including Iran and Pakistan [4-6].

Pakistan is a developing country with no accurate data available on the incidence, prevalence, and mortality rates of inherited blood disorders. However, there are almost 9.8 million β-thalassemia carriers in Pakistan's population with an estimated carrier rate of 5-7% [7]. Another study estimated 8 million carriers of β-thalassemia in Pakistan [8]. A number of factors in Pakistan contribute toward an increase in transfusion-dependent thalassemia, including consanguineous marriages, early marriages, rapid birth rate, and low literacy rate [9].

This study was conducted in a small agricultural district of Pakistan to assess knowledge about thalassemias in families with one or more family members suffering from β-thalassemia major, and to analyze the awareness about treatment and prevention of these diseases in the affected families.

## Materials and methods

We performed our study at the Ali Zaib Foundation in Sahiwal, which is a small agricultural district in Punjab, Pakistan. We collected data from the participants using a direct structured survey method over a period of 30 days in May 2019.

We recruited 100 parents (either a father or a mother) of β-thalassemia major patients in our study by using non-probability, convenient sampling. β-thalassemia patients were diagnosed based on history and Hb electrophoresis with quantification of Hb F and Hb A2 by high-performance liquid chromatography. The objectives of the study were explained to all participants and they were informed that their participation is voluntary. Written informed consent was obtained from the parents. Data were collected via a predesigned questionnaire.

IBM Statistical Package for Social Sciences (SPSS) version 24 (IBM Corp, Armonk, NY) and Microsoft Excel 2014 were the primary tools used to determine frequencies of responses to various questions.

## Results

Our data showed that the prevalence of thalassemia in males was slightly more than in females; 62 patients were males while 38 were females, with a mean age of 8.5 ± 6.2 years. Out of the 100, 43 parents (43%) were illiterate and 8 parents (8%) were highly educated. Sixty-six (66%) parents had consanguineous marriages. Out of 100 participants, 82 belonged to the lower-middle class socioeconomic group (monthly earnings less than 5,000 Pakistani rupees).

Eighty-two participants (82%) were aware of thalassemia through their healthcare professionals and 80% knew that thalassemia is a blood disorder, as shown in Table 1. Seventy-two participants (72%) mentioned cousin marriages as the primary risk factor, while 13 participants (13%) did not know about any risk factor.

**Table 1:** Participants' knowledge about which system is mostly affected by thalassemia.

Table 1: Participants’ knowledge about which body system is affected by thalassemia			
System affected		Frequency (n)	Percentage
	Digestive system	3	3.0
	Urinary system	4	4.0
	Blood	80	80.0
	No idea	13	13.0
	Total	100	100.0

As shown in Table 2, 56 participants were aware that thalassemia is a genetic disorder while 44 participants knew that thalassemia is inheritable. Seventy-six families (76%) had knowledge about the need for prenatal diagnosis in patients with thalassemia. Among those, all of them believed that a prenatal diagnosis of thalassemia was beneficial.

**Table 2:** Participants’ understanding regarding the mode of transmission of thalassemia whether it is hereditary or non-hereditary.

Table 2: Participants’ understanding regarding the mode of transmission of thalassemia			
Mode of transmission		Frequency (n)	Percentage
	By sexual contact	12	12.0
	By blood	8	8.0
	By digestive system	6	6.0
	By hereditary	56	56.0
	No idea	18	18.0
	Total	100	100.0

Unfortunately, only 52 participants (52%) showed understanding about the treatment for thalassemia; all 52 believed that thalassemia is fatal without treatment, while 51 out of 52 participants knew about the importance of adherence to treatment. Sixty participants (60%) had knowledge about the treatment options (including blood transfusions, iron chelation, and bone marrow transplant).

All participants were receiving blood from registered blood banks; however, only 80 (80%) were aware that blood screening is required before every blood transfusion. Only 14% of participants reported that their affected children were receiving iron chelation adequately, while 38% received intravenous chelation only at the time of blood transfusions.

Among the participants, 78 parents (78%) had some knowledge about the prevention of thalassemia. Table 3 shows that 18% parents underwent diagnostic testing before marriage, 44% parents after marriage, and 38% parents did not undergo any testing. All parents (100%) believed that there should be more thalassemia public awareness programs, and that premarital screening should be mandatory.

**Table 3:** Diagnostic testing of the parents

Table 3: Diagnostic testing of the parents			
Diagnostic test done (e.g. complete blood count, hemoglobin electrophoresis)		Frequency (n)	Percentage
	Before marriage (pre-marital testing)	18	18.0
	After marriage	44	44.0
	Undiagnosed – participants who did not undergo any diagnostic test	38	38.0
	Total	100	100.0

## Discussion

Thalassemias are inherited blood disorders characterized by reduced Hb production. There are two main types of thalassemias, alpha (α) thalassemia and beta (β) thalassemia. Our study included 100 parents (either the mother or father) of patients suffering from β-thalassemia. The objective of our study was to assess the knowledge about these disorders among the affected families; it showed that 82% of the parents were aware of the diagnosis of thalassemia while 80% were aware that thalassemia is a blood disorder. In addition, 72% of parents identified consanguineous marriage as a primary risk factor for β-thalassemia, a higher percentage compared to that of a study done in Karachi, Pakistan, where only 12% knew that consanguinity is a risk factor [10]. Fifty-six (56%) of parents were aware that β-thalassemia is hereditary while 64% understood that β-thalassemias are capable of being inherited from parents and then passing it to their children. Our study showed that 66% of patients were born to consanguineous parents, relatively lower than another study conducted in Pakistan [11].

Regarding treatment and prevention of thalassemias, 52% of participants were aware of the importance of treatment for these disorders and understood that delaying treatment can be fatal. Among these 52 participants, 99% were aware of the importance of adhering to the treatment. Surprisingly, 80% of families understood the importance of blood screening prior to blood transfusion, which was remarkably high compared to a study done in Karachi, Pakistan, where only 15.8% of participants knew about the importance of blood screening prior to the transfusions. Only 14% were receiving oral iron chelation therapy, which is not significantly different from the study done in Karachi, Pakistan [10].

In our study, 76% of families had knowledge about the importance of prenatal diagnosis in patients with thalassemias and 18% of the families underwent diagnostic testing for thalassemia before marriage. A study in Pakistan reported an understanding of premarital screening and prenatal diagnosis among 84.3% and 76.5% parents of β-thalassemia major patients [11]. Given the religious and conservative demographics of Pakistan, where therapeutic abortion is considered immoral, premarital screening should be preferred over prenatal tests (chorionic villus sampling) to prevent cases of thalassemia. Our country can follow the screening policies of Cyprus and Greece, which have shown a remarkable reduction in the rate of thalassemias, to identify carriers of thalassemia traits [12,13].

According to a study in Iran, a developing country, the life expectancy of thalassemia patients has increased due to national planning and specialized care but knowledge of the exact rate and age distribution of inherited blood disorders is limited [14]. A study in Jordan revealed that 75% of the families were unaware of screening for thalassemias before having their first child with thalassemia [15]. Pakistani adults have shown inadequate awareness of the risk of thalassemia trait, leading to the birth of a child with thalassemia major [16]. Another study revealed that 60% of families after the birth of a child with thalassemia did not request prenatal diagnosis, mainly due to lack of awareness, difficult access, and high cost [17]. The same study also showed that an increase in maternal education leads to a significant increase in the utilization of prenatal testing [17]. These studies show that the standard of information regarding carrier screening and prenatal diagnosis by the professionals requires substantial improvement.

Limitations

The study included β-thalassemia patients from one thalassemia center, which limits the generalizability of our results. In the future, a large-scale study consisting of patients from different provinces will yield more universal responses and will help to understand the general pattern of behaviors common among thalassemia patients/families. Fortunately, participants were cooperative, and non-response bias was non-existent.

## Conclusions

Our study identified that awareness regarding β-thalassemia and its treatment and prevention is fair but far from ideal with there significant room for improvement. As a measure to prevent thalassemias, the public should be informed and encouraged to undergo voluntary screening to detect thalassemia carriers. Families that are thalassemia carriers should receive genetic counselling and testing. An effort to educate mothers about prenatal diagnosis is warranted. Finally, government and non-government agencies should organize public campaigns to encourage premarital screening and discourage consanguineous marriages.
